# Metronidazole Delivery Nanosystem Able To Reduce the Pathogenicity of Bacteria in Colorectal Infection

**DOI:** 10.1021/acs.biomac.2c00186

**Published:** 2022-05-27

**Authors:** Ana Oliveira, Ana Araújo, Luísa C. Rodrigues, Catarina S. Silva, Rui L. Reis, Nuno M. Neves, Pedro Leão, Albino Martins

**Affiliations:** † 3B’s Research Group, I3Bs − Research Institute on Biomaterials, Biodegradables & Biomimetics of University of Minho, Headquarters of the European Institute of Excellence on Tissue Engineering & Regenerative Medicine, AvePark - Parque de Ciência e Tecnologia, Zona Industrial da Gandra, Barco, Guimarães 4805-017 Portugal; ‡ Life and Health Sciences Research Institute (ICVS), School of Medicine, University of Minho, Campus of Gualtar, Braga 4710-057, Portugal; § ICVS/3B’s − PT Government Associate Laboratory, Braga/Guimarães 4710-057, Portugal

## Abstract

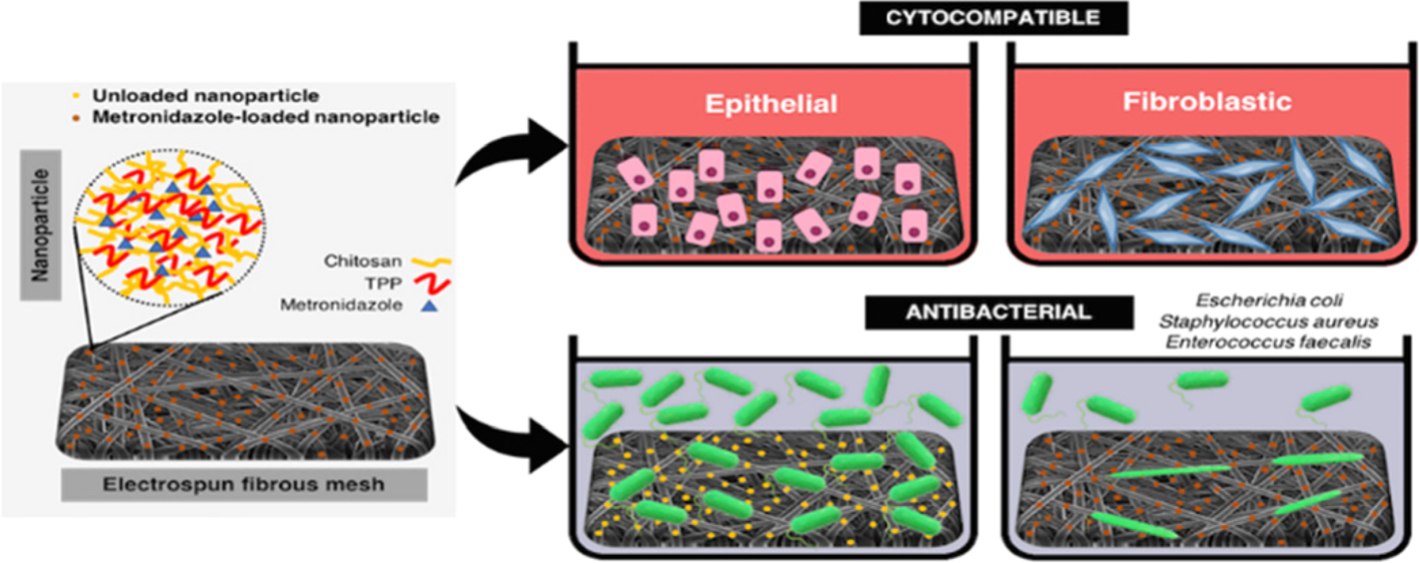

Metronidazole (MTZ) is a drug potentially used for the treatment of intestinal infections, namely, the ones caused by colorectal surgery. The traditional routes of administration decrease its local effectiveness and present off-site effects. To circumvent such limitations, herein a drug delivery system (DDS) based on MTZ-loaded nanoparticles (NPs) immobilized at the surface of electrospun fibrous meshes is proposed. MTZ at different concentrations (1, 2, 5, and 10 mg mL^–1^) was loaded into chitosan–sodium tripolyphosphate NPs. The MTZ loaded into NPs at the highest concentration showed a quick release in the first 12 h, followed by a gradual release. This DDS was not toxic to human colonic cells. When tested against different bacterial strains, a significant reduction of *Escherichia coli* and *Staphylococcus aureus* was observed, but no effect was found against *Enterococcus faecalis*. Therefore, this DDS offers high potential to locally prevent the occurrence of infections after colorectal anastomosis.

## 1 Introduction

Anastomotic leakage after colorectal surgery is a dreaded complication that can reach an incidence rate of 15% in the colon and 28% in the rectum.^1,2^ Several factors have been identified as possible predictors of anastomotic complications, namely, the genetic predispositions of the patients and the characteristics of the surgical procedure that impair the union of the bowel segments.^3,4^ The consequences of an ineffective colorectal anastomosis can be very devastating, and late diagnosis can even lead to cases of generalized peritonitis, compromising the patients’ lives.^5^

The human intestine is composed of a high diversity of microorganisms that act in symbiosis with intestinal cells to maintain the normal environment, *Escherichia coli* and *Enterococcus faecalis* being some of the typical bacteria.^6^ Despite this, the intestinal microbiota may play a significant role in infectious events after colorectal anastomosis. The intestinal flora close to the anastomosis site can interact with intestinal tissue and impair anastomotic healing. The surgical tissue damage and the tension at the anastomotic line promote the phenotypic transformation of intraluminal microbes, giving them pathogenicity.^3,4^ Fecal content analysis demonstrated significant changes in the intestinal environment after surgery, including an increase in pathogenic bacteria.^7^*Escherichia coli* is the main enteric Gram-negative rod in the colon^8^ and the most abundant after the completion of an anastomosis,^8,9^ representing 94% of the microorganisms found.^9^ Although the colon lumen is the biggest source of contamination, colorectal surgery is also widely influenced by infections of the surgical site promoted by bacteria on the skin and from the environment, such as *Staphylococcus aureus*,^8^ and therefore, their potential to cause postoperative disturbances should not be discarded.

Different approaches have been proposed to tackle the problems that may occur after a colorectal anastomosis. If, on the one hand, there are attempts on mechanical reinforcements to avoid tension at the anastomotic line,^10^ there are also strategies based on the administration of therapeutic drugs to reduce the inflammatory mechanisms and the bacterial load at the anastomotic region.^4,11^ Metronidazole (MTZ) is a potential drug used for the treatment of infections related to inflammatory disorders of the gastrointestinal tract due to its amebicidal, antiprotozoal, and antibiotic action on a wide spectrum of anaerobic microorganisms.^12−14^ This drug enters bacteria by passive diffusion as a prodrug, being activated in the cytoplasm. Through intracellular reduction, MTZ is converted into a cytotoxic nitrous free radical that inhibits synthesis and promotes DNA damage by oxidation, breaking it into a single strand. Consequently, the DNA is degraded, causing the death of bacteria.^15^ However, after oral or intravenous administration, in addition to the gastrointestinal tract, high concentrations of the drug are also found in the liver, bladder, kidneys, and vagina.^13^ Thus, beyond the unwanted side effects, a reduced amount reaches the desired colorectal region. Particularly, when the drug is administered orally it is prematurely degraded before reaching the colorectal environment due to the acidic pH of the stomach and the presence of various degradative enzymes.^16^

Colon drug delivery systems (DDSs) acquired increasing interest because they avoid the degradation of drugs by the gastric pH, allowing the selective release of the drug in the colonic environment and, consequently, the reduction of undesirable side effects.^17^ Colloidal drug carriers, e.g., polymeric nanoparticles (NPs), are among the several DDSs that have been studied to prevent systemic drug distribution. NPs made of chitosan (CH), a natural cationic polysaccharide derived from the partial deacetylation of chitin, offer the possibility of controlling the drug release rate to specific sites, in addition to their biocompatibility, nontoxicity, biodegradation by the bacterial flora of the colon, and mucoadhesive properties to the negatively charged luminal surface of the colon.^18,19^ Based on its properties, MTZ-loaded CH NPs have been proposed for colonic applications.^19−22^ However, there is no guarantee that such NPs reach the intervened colorectal region being confined to the anastomotic site. On the other side, electrospun fibrous meshes (eFMs) are polymeric substrates with excellent mechanical properties and surface characteristics that make them interesting in the biomedical field.^23−25^ These substrates present high specific surface area, flexibility in the surface functionality, physical fibrous structure that mimics the morphology of the native extracellular matrix of most soft tissues, and porosity that allows the ingrowth of cells. These substrates become even more attractive because they allow the modification of their surface to be combined with delivery systems such as cationic CH NPs.^26^ This possibility allows the drug release from the NPs to be more sustained and directed to the specific site, increasing the therapeutic efficacy.

Taking advantage of these functionalities, this work proposes a nanosystem based on MTZ-loaded NPs immobilized at the surface of eFMs to be applied directly at the anastomotic site, aspiring to control spatially and temporally the release of MTZ in the colorectal environment, avoiding off-target side effects. After cytocompatibility screening assays of a wide range of MTZ concentrations loaded into NPs immobilized at the surface of eFMs, the antibacterial properties of the best formulation were tested for *Escherichia coli*, *Staphylococcus aureus*, and *Enterococcus faecalis*.

## 2 Materials and Methods

### 2.1 Materials

Polycaprolactone (PCL) (*M*_n_ = 70,000–90,000 determined by gel permeation chromatography (GPC), MTZ (certified reference material), sodium tripolyphosphate (TPP), and d-(+)-glucose were purchased from Sigma-Aldrich. CH 95/20 (*M*_w_ = 40–150 kDa by GPC) was purchased from Heppe Medical Chitosan GmbH. Chloroform, *N*,*N*-dimethylformamide (DMF), and acetic acid were purchased from Honeywell Specialty Chemicals Seelze GmbH. Centrifugal concentrators Vivaspin 20 (300 000 MWCO, PES) were purchased from Fisher Scientific. Dialysis membranes (50 kD) were purchased from Spectrum Laboratories. CellTiter 96 AQueous One Solution was purchased from Promega Corporation. Human colon epithelial (CCD 841 CoN, ATCC CRL-1790) and fibroblastic (CCD-18Co, ATCC CRL-1459) cell lines, Eagle’s Minimum Essential Medium (EMEM), and *Staphylococcus aureus* (*S. aureus*, ATCC 25923), *Escherichia coli* (*E. coli*, ATCC 25922), and *Enterococcus faecalis* (*E. faecalis*, ATCC 29212) were obtained from the American Type Culture Collection. Fetal bovine serum (FBS), antibiotic antimycotic solution, and TrypLE Express were purchased from Life Technologies Europe BV. Tryptic soy agar (TSA) and tryptic soy broth (TSB) were purchased from Liofilchem.

### 2.2 Optimization of the NPs Production Process

CH NPs were produced by the ionic gelation technique induced by TPP as described elsewhere,^19^ with some adjustments. Briefly, CH was dissolved in a 1% (v/v) acetic acid solution and TPP was dissolved in ultrapure water at different concentrations (Table 1). Using a precision syringe pump (FUSION 200, KR Analytical), the TPP solution was added dropwise to the CH solution and stirred for 1 h at 990 rpm. Then, NPs were collected using Vivaspin filters for 20 min at 4000 rpm. In order to avoid the NPs aggregation, glucose at 2 mg mL^–1^ was added in the centrifugation step, acting as a stabilizing agent that promotes the creation of a protective layer of hydration around the CH chains.^27,28^ Finally, NPs were filtered with 0.45 μm filters. Different CH:TPP ratios, pH adjustments, and CH and TPP concentrations were optimized as described in Table 1 and characterized to achieve the best formulation (F) of NPs identified in bold.

**Table 1:** Optimized Parameters in the NPs Production Process

F	solution concentration (w/v)		volume ratio (CH:TPP)	ionic molar ratio (CH:TPP)	pH		
	CH	TPP			CH	TPP	before the centrifugation step
1	0.3%	0.1%	1:1	1.5:1			
2			2:1	3:1			
3			1:2	0.7:1			
**4**	**0.3%**	**0.1%**	**1:1**	**1.5:1**	**4.5**		
5				1.5:1	4.5	4.5	
6	0.3%	0.1%	1:1	1.5:1			4.5
7				1.5:1			6
8	0.2%	0.1%	1:1	1:1	4.5		
9	0.4%			2:1			
10	0.3%	0.05%	1:1	3:1	4.5		
11		0.2%		0.7:1			

### 2.3 Preparation of MTZ-Loaded NPs

MTZ-loaded NPs were produced using the protocol described above, keeping the concentration of the CH solution at 0.3% (w/v) and the TPP solution at 0.1% (w/v) with a volume ratio of 1:1 (CH/TPP). Only the pH of the CH solution was adjusted to 4.5 (F4). Then, MTZ was dissolved in the CH solution, leading to different concentrations, i.e., 1, 2, 5, and 10 mg mL^–1^, after the addition of TPP.

### 2.4 Characterization and Stability of NPs

The size and polydispersity index (PDI) of NPs in ultrapure water were determined by dynamic light scattering using a Zetasizer (Nano ZS, Malvern). The size analysis was performed at 25 °C. Zeta potential measurements were also performed using a dip cell in automatic mode. The stability (size and PDI) of the different formulations, i.e., unloaded-NPs (CTR-NPs), NPs with 1 mg mL^–1^ MTZ (MTZ1-NPs), NPs with 2 mg mL^–1^ MTZ (MTZ2-NPs), NPs with 5 mg mL^–1^ MTZ (MTZ5-NPs), and NPs with 10 mg mL^–1^ MTZ (MTZ10-NPs), under storage conditions (ultrapure water) was also evaluated along 91 days.

### 2.5 Drug Entrapment Efficiency

The entrapment efficiency (EE) of MTZ into the NPs was calculated by the measurement of the initial and the nonencapsulated MTZ concentrations, according to the following formula:
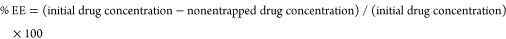


To obtain the nonentrapped drug concentration, the supernatant obtained after NPs centrifugation was used. The absorbance of the samples was analyzed by standard UV spectroscopy (UV 1601, Shimadzu) at the wavelength 319.5 nm. For the calculations, the absorbance of the supernatant of CTR-NPs was subtracted from the values of the supernatant of all MTZ-NPs conditions.

### 2.6 Morphological Characterization of NPs

Scanning electron microscopy (SEM) was used for the morphological characterization of NPs. For this, the NPs solution was spread on a glass slide and then dried at room temperature. Using a Leica Sputter Coater EM ACE600, the dried NPs were coated with platinum and then analyzed using a high-resolution field emission scanning electron microscope with focused ion beam (AURIGA COMPACT, ZEISS).

### 2.7 Production of the eFMs

The production of PCL eFMs was performed as described elsewhere.^29^ A 20% (w/v) PCL solution was prepared with an organic mixture of chloroform and DMF at a 8:2 volume ratio. This solution was electrospun at 15.5 kV, using a needle tip with a distance of 20 cm to ground collector, and a flow rate of 1.0 mL h^–1^. After dispersing 1 mL of PCL solution, the eFM was allowed to dry for 1 day.

### 2.8 Surface Activation of the eFMs

For the introduction of oxygen-containing groups at the surface of the fibers, eFMs were activated by oxygen plasma as described elsewhere.^29^ Briefly, the PCL eFMs were placed in the chamber of a low-pressure plasma system (Zepto, Diener Electronic), subjected to vacuum (<0.4 mbar), and then filled with oxygen. The pressure of the plasma chamber was maintained near 1 mbar during the treatment. A power intensity of 30 W was applied for 5 min.

### 2.9 Immobilization of NPs at the Surface of eFMs

For the NPs immobilization, the produced NPs solution was incubated for 20 h at 4 °C on both sides of the activated eFM based on previous work.^26^ After this time period, the NPs solution was removed and eFMs were allowed to dry at 4 °C.

#### 2.9.1 Chemical Characterization of eFMs

The eFM, eFM activated with oxygen plasma treatment (eFM−), and eFM immobilizing NPs (eFM + CTR-NPs) were characterized by X-ray photoelectron spectroscopy (XPS). A Kratos Axis-Supra instrument equipped with an aluminum Kα (Al-Kα) monochromatized radiation X-ray source (1486.6 eV) and ESCApe software was used to perform the analysis of the samples. All the measurements were performed in a vacuum of at least 10^–7^ Pa and a take-off angle of 90° relative to the sample surface, to collect the photoelectrons. The superficial chemical composition of samples was assessed using a Constant Analyzer Energy mode with an emission current of 15 mA and pass energies of 160 and 40 eV for survey and high-resolution spectra, respectively. The high-resolution region analysis was performed for C 1s, O 1s, and N 1s, using a linear-type background to its fitting. The lower binding energy C 1s photo peak, which is related to the C 1s hydrocarbon peak, was set at 285.0 eV for charge referencing. Data are presented as average ± standard deviation (SD).

#### 2.9.2 Quantification of the Immobilization Capacity

The immobilization capacity of NPs at the surface of eFMs was quantified using Zetasizer (Nano ZS, Malvern) equipment. For this, the position, attenuator, and counting rate of the initial NPs solution were determined. After removing the NPs solution from the immobilization step at eFMs, the counting rate of the nonimmobilized NPs solution was determined, fixing the attenuator and the position of the initial NPs solution. For the calculations, a 100% immobilization was considered for the counting rate of the initial NPs solution.

#### 2.9.3 Morphological Characterization of the Nanosystem

The morphologies and distribution of immobilized NPs were characterized using a high-resolution field emission scanning electron microscope with focused ion beam (AURIGA COMPACT, ZEISS) and compared with eFM and eFM–. Before this analysis, the samples were coated with platinum using a Leica Sputter Coater EM ACE600.

### 2.10 Contact Angle Measurements

The static contact angles of eFM, eFM–, and eFM + CTR-NPs were measured using a goniometer (OCA 15PLUS, DataPhysics Instruments) at room temperature. Using the sessile drop method, 3 μL of glycerol was dispensed through a motor-driven syringe at the surface of the samples. At least two measurements of three independent experiments were performed. Data are presented as average ± SD.

### 2.11 Cytocompatibility Evaluation of the MTZ-Loaded NPs Immobilized at the Surface of eFMs

Two colon cell lines, CCD 841 CoN (epithelial morphology) and CCD-18Co (fibroblastic morphology), were used to test the cytocompatibility of CTR-NPs and a range of MTZ-NPs immobilized at the surface of eFMs. All conditions were tested with 5 mm diameter disks of eFMs.

#### 2.11.1 Cell Culture and Seeding

Both cell lines grew in EMEM with 10% (v/v) of FBS and 1% (v/v) of antibiotic antimycotic solution. They were maintained at 37 °C in a humidified atmosphere of 5% CO_2_. CCD 841 CoN and CCD-18Co cells were used at passages 13–16. Cell seeding was performed by dropping 50,000 cells resuspended in the culture medium over each sample. The cells were allowed to adhere for 4 h, and then, the culture medium was added. Unloaded NPs immobilized at the surface of the eFM (eFM + CTR-NPs) were used as controls.

#### 2.11.2 Cell Viability

The metabolic activity of CCD 841 CoN and CCD-18Co cells seeded on loaded and unloaded NPs immobilized at the surface of the eFM was determined by the MTS assay. After 1, 3, and 7 days, the culture medium was removed and the samples were rinsed with Dulbecco’s phosphate-buffered saline solution. Then, a mixture of DMEM without phenol red and 1% (v/v) of antibiotic antimycotic solution and the MTS reagent, at a 5:1 volume ratio, was added to each sample and left to incubate for 3 h. The absorbance of the MTS reaction medium from each sample was read in triplicate at 490 nm (Synergy HT, BioTEK).

#### 2.11.3 Morphological Characterization

The samples were washed with a phosphate-buffered saline (PBS) solution and, then, fixed with a 2.5% (v/v) glutaraldehyde solution. After this, they were washed again with a PBS solution and dehydrated using successive solutions of 50, 70, 90, and 100% ethanol (twice, 15 min each). The samples were left to dry. Thereafter, the samples were coated with gold using a Cressington Sputter Coater 108A. The morphology of the cells in the samples was observed using a scanning electron microscope (JSM-6010 LV, JEOL).

### 2.12 MTZ Release from NPs Immobilized at the Surface of the eFM

The release of MTZ from NPs immobilized at the surface of the eFM was studied using a dialysis method. eFM disks with a 5 mm diameter previously treated with oxygen plasma and immobilizing NPs loaded with the highest concentration of MTZ (i.e., eFM + MTZ10-NPs) were placed inside dialysis membranes and filled with a 0.9% (w/v) NaCl solution. The ends of the dialysis membranes were closed with Nylon thread, tested for leakage, and fully immersed in 5 mL of 0.9% (w/v) NaCl solution at pH 7.4, as the release medium. The release study was conducted at 37 °C and 60 rpm for 156 h. At each time point, an aliquot of 500 μL was collected and replaced with fresh NaCl solution. The absorbance was measured by standard UV spectroscopy (UV-1601, Shimadzu) at 319.5 nm. The MTZ concentration of each sample was calculated using the calibration profile based on the absorbances of free MTZ solutions in NaCl of known concentrations at the same wavelength. To determine the mechanism of MTZ release, the results obtained were fitted with several release kinetic mathematical models (e.g., zero-order, first-order, Higuchi, and Korsmeyer–Peppas). The best model was selected based on the value of the correlation coefficient (*R*^2^).

### 2.13 Antimicrobial Activity Assays

The susceptibility of Gram-negative bacteria (*E. coli*) and Gram-positive bacteria (*S. aureus* and *E. faecalis*) against the eFM immobilizing MTZ10-NPs in anaerobic conditions was tested. The bacterial viability in the liquid medium was assessed through colony-forming units (CFUs) count, absorbance measurement, and morphological characterization by SEM. As the sample control, eFMs immobilizing CTR-NPs were used. Both conditions were performed with 5 mm diameter disks of eFMs.

#### 2.13.1 Bacterial Cultivation and Inoculum Preparation

*E. faecalis*, *E. coli*, and *S. aureus* subcultures were incubated at 37 °C for 18–24 h on TSA. For the preparation of suspension cultures, single colonies were removed from the agar plate, inoculated in 10 mL of TSB, and incubated for 24 h at 37 °C under shaking conditions at 120 rpm. After incubation, the absorbance at 600 nm was read using a spectrophotometer (Genesys 30, Thermo Fisher Scientific) and adjusted to that of a McFarland 0.5 turbidity standard, corresponding to 1–2 × 10^8^ CFUs mL^–1^. Then, the concentration was adjusted to 1–2 × 10^6^ CFUs mL^–1^.

#### 2.13.2 Bacterial Viability in the Liquid Medium by Viable Counts and Absorbance Measurement

The eFM disks with CTR-NPs or MTZ10-NPs were immersed in 200 μL of TSB inoculated with 1–2 × 10^6^ CFUs mL^–1^ onto a 96-well plate and incubated at 37 °C with agitation (150 rpm) for 24 h under anaerobic conditions. For this, an anaerobic incubation jar (Sigma-Aldrich) with anaerobic atmosphere generation bags (Sigma-Aldrich) and a dry anaerobic indicator strip (Millipore) was set. Afterward, the CFUs mL^–1^ counted by serial dilutions, and each bacterial suspension was spread onto TSA plates and incubated overnight at 37 °C. In addition to the sample control constituted of the eFM immobilizing CTR-NPs, each bacterial suspension in TSB was used as a positive control. Additionally, the absorbance at 600 nm was measured using a microplate reader (Synergy HT, BioTEK). For the absorbance measurement, additional negative controls made under the same conditions were used, i.e., TSB without bacterial inoculation and samples, and TSB containing the eFM immobilizing CTR-NPs or MTZ10-NPs without bacterial inoculation. Three independent assays were conducted, and each condition was tested in triplicate.

#### 2.13.3 Morphological Characterization of Bacteria by SEM

The morphology of eFM disks immobilizing CTR-NPs or MTZ10-NPs was also assessed by SEM after 24 h of inoculation with the bacterial suspensions (1–2 × 10^6^ CFUs mL^–1^) at 37 °C and 150 rpm under anaerobic conditions. After incubation, the culture medium was removed, and the samples were washed with PBS and then fixed with a 2.5% (v/v) glutaraldehyde solution in PBS. After this, samples were washed with a PBS solution and dehydrated as described in 2.11.3. The samples were left to dry for 1 day. Thereafter, the samples were coated with platinum using a Leica Sputter Coater EM ACE600. The morphology of adhered bacteria was observed in a High-Resolution Field Emission Scanning Electron Microscope with Focused Ion Beam (AURIGA COMPACT, ZEISS).

### 2.14 Statistical Analysis

Statistical analysis was performed using Graph Pad Prism Software. Differences between conditions were analyzed using nonparametric tests (Kruskal–Wallis or Mann–Whitney tests), and *p* < 0.01 was considered significant. For multiple comparisons, Dunn’s test was used.

## 3 Results

### 3.1 Optimization of the NPs Production Process and Morphological Characterization

**Figure 1:**
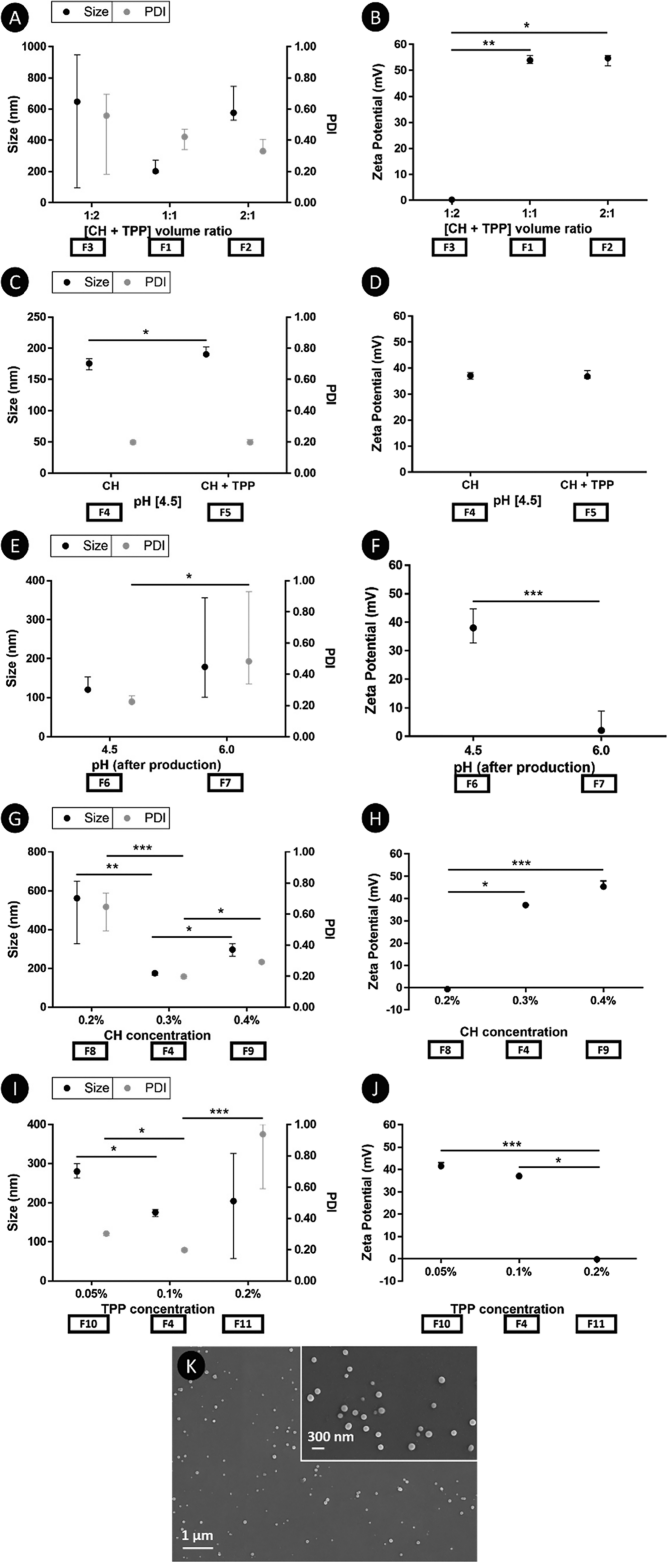
NPs’ size, PDI, and zeta potential of different formulations: (A,B) evaluation of CH and TPP solutions volumes ratios in the final formulation, not adjusting the pH of any solution; (C,D) evaluation of pH adjustment of CH and TPP solutions to 4.5, keeping the CH concentration at 0.3%, TPP at 0.1%, and the volume ratio of CH:TPP at 1:1; (E,F) evaluation of pH adjustment after NPs production, keeping the CH concentration at 0.3%, TPP at 0.1%, and the volume ratio of CH:TPP at 1:1; (G,H) evaluation of CH concentration adjustment, keeping the TPP concentration at 0.1%, the volume ratio of CH:TPP at 1:1, and the pH of CH at 4.5; (I,J) evaluation of TPP concentration adjustment, keeping the CH concentration at 0.3%, the volume ratio of CH:TPP at 1:1, and the pH of CH at 4.5; (K) morphological characterization of the best NPs formulation (F4) by SEM. Data are presented as median ± interquartile range (i. q. r.). Results were considered statistically significant at *p* < 0.01 (*); ** *p* < 0.001; and *** *p* < 0.0001.

The process of NPs production was optimized in terms of CH and TPP solutions volumes ratios, pH of the CH and TPP solutions or after NPs production, and concentration of the CH and TPP solutions. The results obtained showed that when TPP was in excess in the final formulation (F3), the NPs presented a polydisperse size (Figure 1A). For the 2:1 volume ratio (F2), although less polydisperse, the NPs still have a higher size. Although the differences were not statistically significant, the best formulation was obtained at a 1:1 volume ratio of CH:TPP. Therefore, this volume ratio was used for all further experiments. In terms of zeta potential (Figure 1B), it was found that when a higher proportion of TPP was used, the NPs had a neutral charge differing from the other ratios, which had a positive charge (*p* < 0.01 and *p* < 0.001 for F2 and F1, respectively). Also, the pH adjustment of the CH and TPP solutions before the production of the NPs was evaluated (Figure 1C). Adjusting only the pH of the CH solution to 4.5 (F4) produced smaller NPs than adjusting both CH and TPP solutions (F5) (*p* < 0.01). No differences were found in the zeta potential (Figure 1D), with NPs presenting a stable positive charge for both conditions. When the pH of the NPs solution after production and before the centrifugation step was adjusted to 6 (F7), the NPs presented a high PDI compared to when the pH was adjusted to 4.5 (F6) (*p* < 0.01) (Figure 1E). The increase of pH from 4.5 to 6 promoted a significant decrease of the NPs zeta potential to a small positive charge (*p* < 0.0001) (Figure 1F). In addition to these parameters, the influence of the CH concentration was also verified, while the TPP concentration was maintained at 0.1%. When the CH concentration was decreased from 0.3% (F4) to 0.2% (F8), the size and PDI of the NPs significantly increased (*p* < 0.001 and *p* < 0.0001, respectively) (Figure 1G) accompanied by a drastic reduction of the NPs charge to slightly negative values (*p* < 0.01) (Figure 1H). In turn, an increase in CH concentration from 0.3% (F4) to 0.4% (F9) promoted a slighter increase in the size and PDI of the NPs (*p* < 0.01), but a positive charge was maintained with significant differences being observed when compared to a 0.2% of CH concentration (F8) (*p* < 0.0001). Finally, the effect of TPP concentration was evaluated by keeping the CH concentration at 0.3%. Both the reduction of the TPP concentration from 0.1% (F4) to 0.05% (F10) and its increase from 0.1% (F4) to 0.2% (F11) promoted an increase in the PDI of the NPs (*p* < 0.01 and *p* < 0.0001, respectively), but only significant differences were found in the first situation due to the increase in the size of the NPs (*p* < 0.01). (Figure 1I). Regarding the zeta potential, significant differences were found when the TPP concentration was increased from 0.05% (F10) or 0.1% (F4) to 0.2% (F11) due to a drastic reduction of the NPs charge to slightly negative values (*p* < 0.0001 and *p* < 0.01, respectively) (Figure 1J).

According to this optimization, for subsequent tests, NPs produced from a 1:1 volume ratio of 0.3% CH at pH 4.5 and 0.1% TPP were used (F4). The morphology of this NPs formulation was characterized by SEM in a dry state. According to Figure 1K, the NPs presented a round shape and a nonaggregated structure.

### 3.2 Properties and Stability of MTZ-loaded NPs

**Figure 2:**
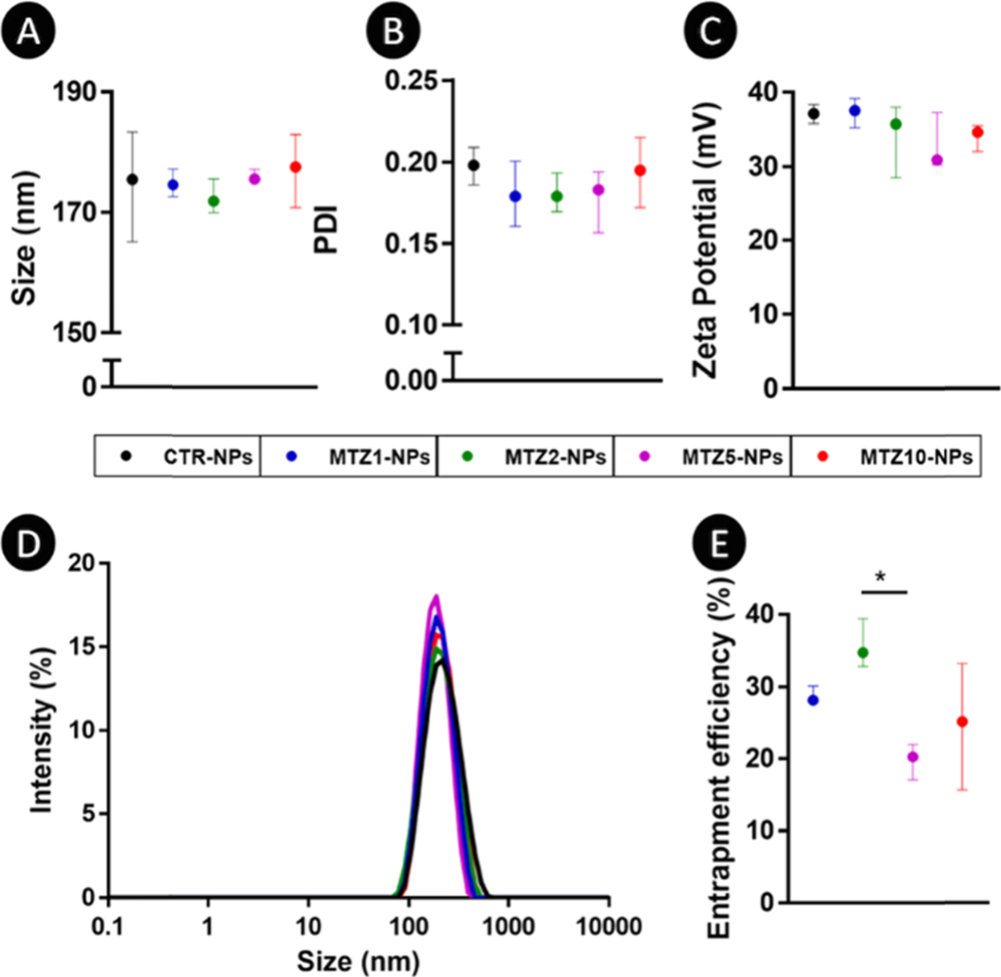
Characterization of CTR-NPs and MTZ-NPs: (A) size; (B) PDI; (C) zeta potential, (D) size distribution representation; and (E) entrapment efficiency (%). Data are presented as median ± i. q. r. Results were considered statistically significant at *p* < 0.01 (*).

**Figure 3:**
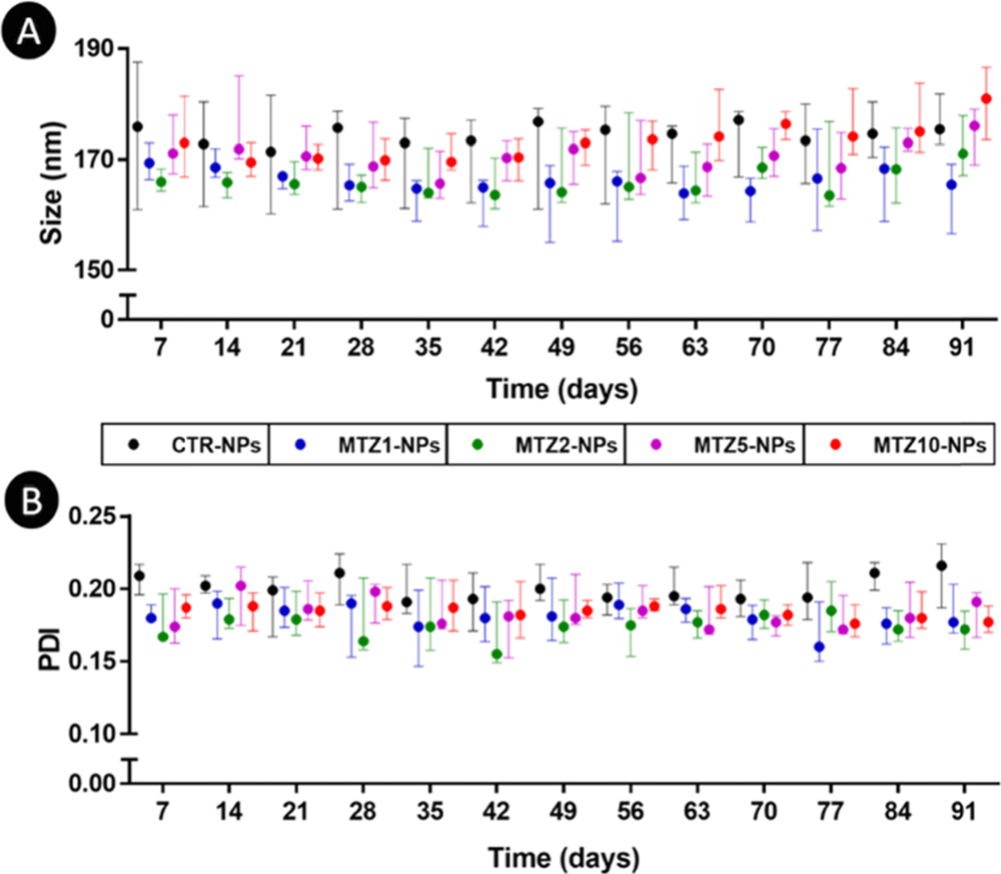
Stability of the different NPs formulations under storage conditions: (A) size and (B) PDI. Data are presented as median ± i. q. r.

For the preparation of MTZ-loaded NPs, a range of MTZ concentrations (i.e., 1, 2, 5, and 10 mg mL^–1^) were tested and compared with CTR-NPs. The results showed that an increase in the concentration of MTZ did not interfere with the size and PDI of the NPs, since any differences were found between conditions (Figure 2,A,B, respectively). Furthermore, the addition of MTZ did not promote statistical differences in the positive zeta potential of NPs along the MTZ concentrations (Figure 2C). A representative image of the size distribution of CTR-NPs and NPs loaded with the different concentrations of MTZ is shown in Figure 2D. The drug EE of all prepared MTZ-loaded NPs was also assessed (Figure 2E), but differences were only found between the MTZ2-NPs and MTZ5-NPs conditions (*p* < 0.01). The properties of the NPs (i.e., size and PDI) were maintained over time (91 days) under storage conditions in ultrapure water (Figure 3,A,B), with the size ranging between 151.6 and 199.5 nm.

### 3.3 NPs Immobilization

**Figure 4:**
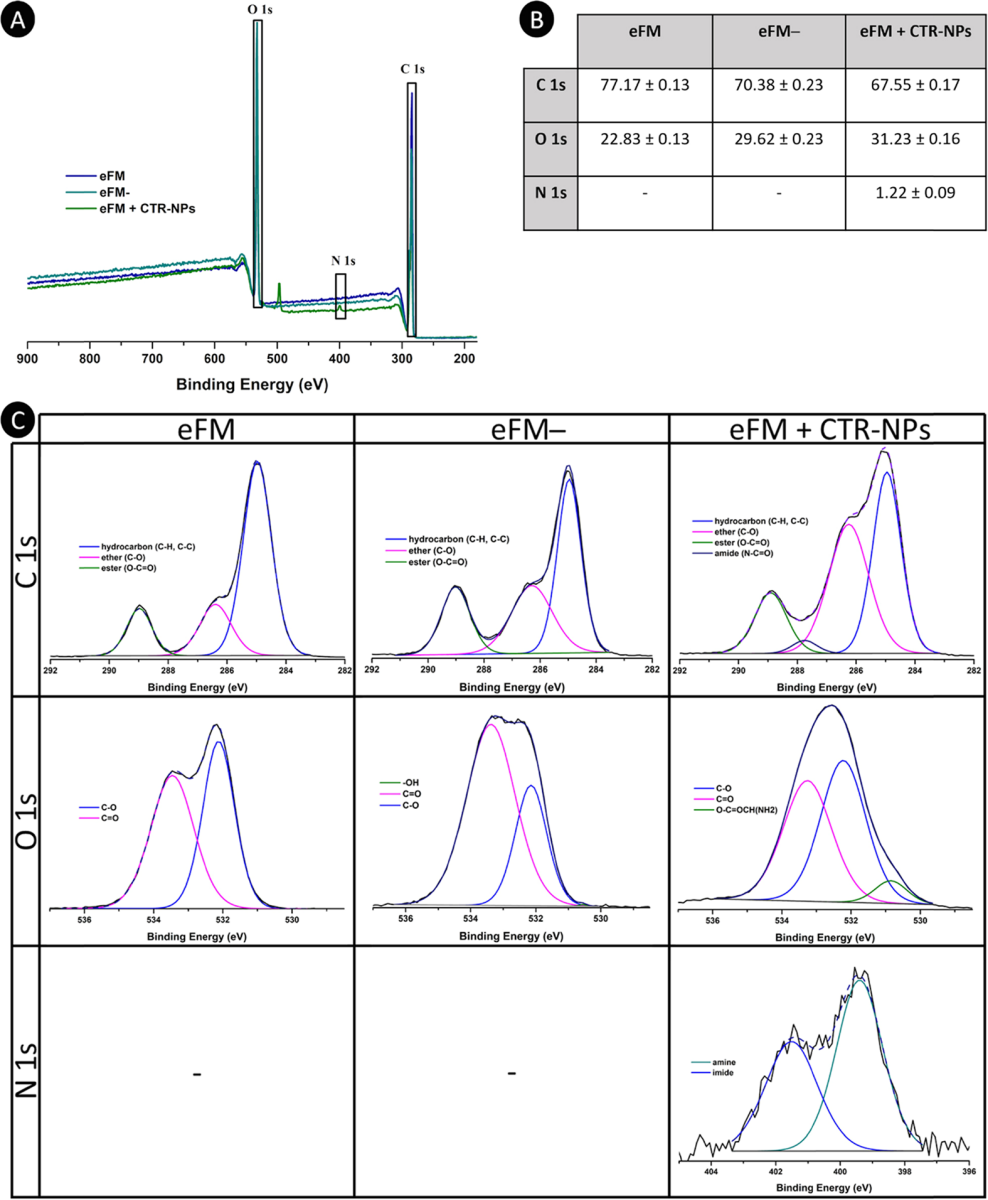
XPS analysis of the eFM, eFM–, and eFM immobilizing NPs (eFM + CTR-NPs): (A) wide scan; (B) elemental composition (% C, O, and N) derived from the region spectrum—data are presented as average ± SD; (C) C 1s, O 1s, and N 1s region scans.

The ability of the plasma-activated eFM to immobilize NPs was also studied. For this, the eFM, eFM–, and eFM + CTR-NPs were analyzed by XPS, as shown in Figure 4A. From the XPS survey spectrum, the elements present at the sample surface were identified, and from the high-resolution region spectra, the atomic ratios (at%) of C, O, and N at the surface of eFMs were calculated (Figure 4B). After oxygen plasma treatments, the O content of the eFM surfaces increased from 22.83 to 29.62 at%. From the XPS O 1s spectra of the eFM and eFM– conditions, it is possible to observe an inversion on the prevalence of the type of oxygen binding after the treatment, with a significant increase in the incidence of C=O species. While the XPS C 1s spectra of the eFM and eFM– conditions remained similar in the overall content, they differed in terms of proportions of carbon environments. After the oxygen plasma treatment, there was an evident increase in the amount of the C band at higher binding energies, which is attributed to the oxidation of the eFM surfaces. When CH NPs were immobilized at the surface of the plasma-activated eFM, the appearance of the nitrogen peak related to the created amide bond was observed. The high-resolution XPS region C 1s spectrum showed significant changes in the carbon environment. The eFM (Figure 4C) C 1s spectrum was fitted allowing one to identify the three components of its chemical structure: the hydrocarbons, ether, and ester groups. When eFMs were activated with oxygen plasma treatment, the intensity of the C=O band increased, and that of C–O reduced, and the appearance of a hydroxyl-group-related band can also be observed. After immobilizing NPs at the surface of the plasma-activated eFM, an additional band appeared in the fitted carbon spectrum, which is related to the new amide bond formation around 287.5 eV. Also, an increase in the intensity of the C–O group band was observed, which is related to the presence of CH NPs. Comparing the O 1s spectra for eFM– and eFM + CTR-NPs, a reduction of the C=O group band was noted, indicating a reduction of these groups at the surface, which may suggest the presence of NPs at the studied locations and give indication of the functional group used in the binding. The N 1s curve that appeared only for eFM + CTR-NPs was fitted using two peaks that could be assigned to the amine (∼399 eV) and imide (∼401 eV) groups, which suggest the intervention of the C=O group in the covalent binding with the nitrogen of the CH amino/acetamido functional group, confirming the binding of NPs to the plasma-activated eFM.

**Figure 5:**
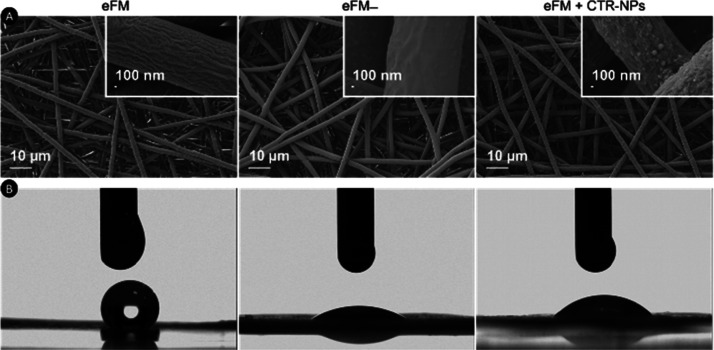
Morphology and hydrophilicity of the eFM, eFM–, and eFM immobilizing NPs (eFM + CTR-NPs): (A) SEM micrographs; (B) representative images of the glycerol contact angle measurements.

The morphological characterization performed by SEM (Figure 5A) showed that the NPs are uniformly distributed at the surface of the fibers. The size of the NPs was small enough to penetrate the pores between the fibers layers, being immobilized not only at the first plane but also in fibers within the eFMs. The percentage of NPs immobilization was determined based on the difference between the counting rates of the NPs’ initial solution and the NPs’ solution after the immobilization. This process showed a median immobilization efficiency of 66.7 (56.5–72.2)%.

As can be observed in Figure 5B, the contact angle measurements showed that eFM surface activation induced a decrease in the value of the glycerol contact angle (eFM: 134.54° ± 0.47°; eFM–: 32.04° ± 0.82°), suggesting that the surfaces became hydrophilic after the oxygen plasma treatment. Interestingly, the immobilization of NPs at the surface of activated eFMs slightly augmented the value of the glycerol contact angle (47.53° ± 5.37°) not affecting greatly the hydrophilicity.

### 3.4 Cytocompatibility Evaluation of eFMs Immobilizing CTR-NPs and Different Formulations of MTZ-NPs

**Figure 6:**
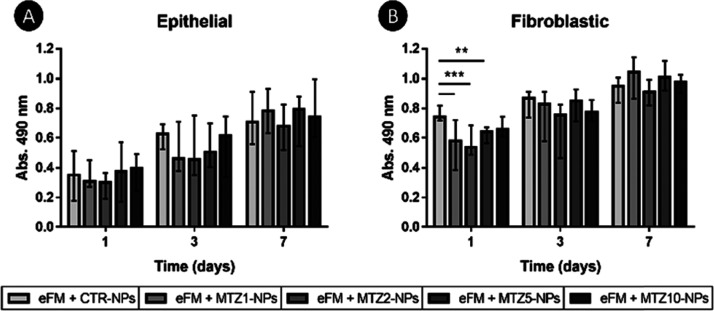
Metabolic activity of human colonic cells cultured on eFMs with CTR-NPs and NPs loaded with different concentrations of MTZ along time points (1, 3, and 7 days): (A) epithelial CCD 841 CoN (B) and fibroblastic CCD-18Co cell lines. Data are presented as median ± i. q. r. Results were considered statistically significant at *p* < 0.01 (*); ** *p* < 0.001; and *** *p* < 0.0001.

The metabolic activity of colon epithelial (CCD 841 CoN cell line) and fibroblastic (CCD-18Co cell line) cells seeded on eFMs immobilizing CTR-NPs or MTZ-NPs was determined by the MTS assay at different time points (1, 3, and 7 days). The results showed that an increase in the concentration of MTZ did not significantly affect the viability of colon epithelial cells (Figure 6A). Only a decrease in fibroblastic cells was observed for the 1^st^ day, at MTZ1, MTZ2, and MTZ5 concentration (Figure 6B). Then, cell viability was recovered for longer time points. The morphology of both cell types cultured on the eFMs immobilizing CTR-NPs or NPs loaded with different concentrations of MTZ was observed by SEM (Figures S1 and S2), presenting similar adherence in all conditions. Since the concentration of MTZ loaded in the NPs immobilized at the surface of the eFM did not interfere with cell viability, the condition with the highest concentration of MTZ into the NPs (eFM + MTZ10-NPs) was used in subsequent tests.

### 3.5 Release Study

**Figure 7:**
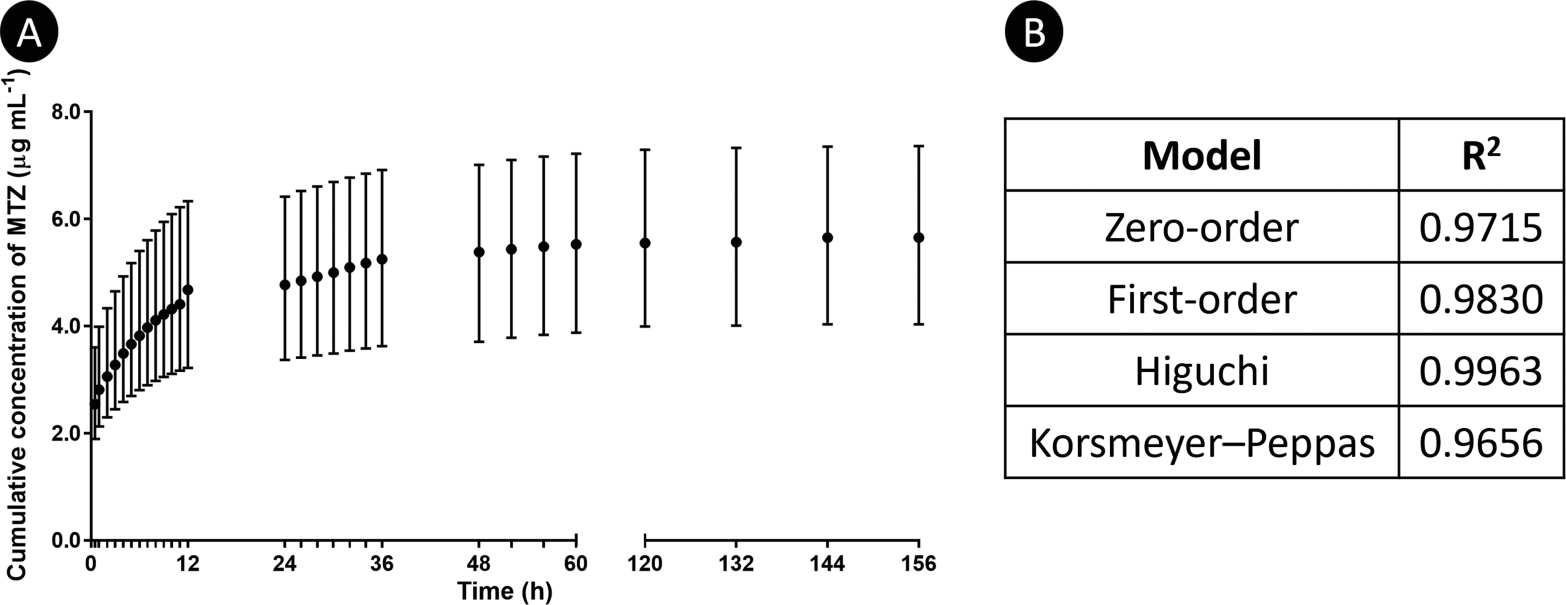
(A) Cumulative release of MTZ from MTZ10-NPs immobilized at the surface of eFMs; data are presented as median ± i. q. r. (B) Calculated *R*^2^ of different kinetic models of drug release.

The release profile of the higher concentration of MTZ upon immobilization on the eFM (i.e., eFM + MTZ10-NPs) was evaluated using a 0.9% NaCl solution as a release medium at 37 °C and 60 rpm to mimic the physiological conditions (Figure 7A). The profile showed a faster release of MTZ during the first 12 h followed by a slower and gradual release until the end of the study. The results obtained were fitted to various kinetic models, such as zero-order, first-order, Higuchi, and Korsmeyer–Peppas, and the corresponding *R*^2^ values are presented in Figure 7B. Data indicated that Higuchi was the best model to fit the MTZ release from eFM-immobilized NPs.

### 3.6 Antimicrobial Assays

**Figure 8:**
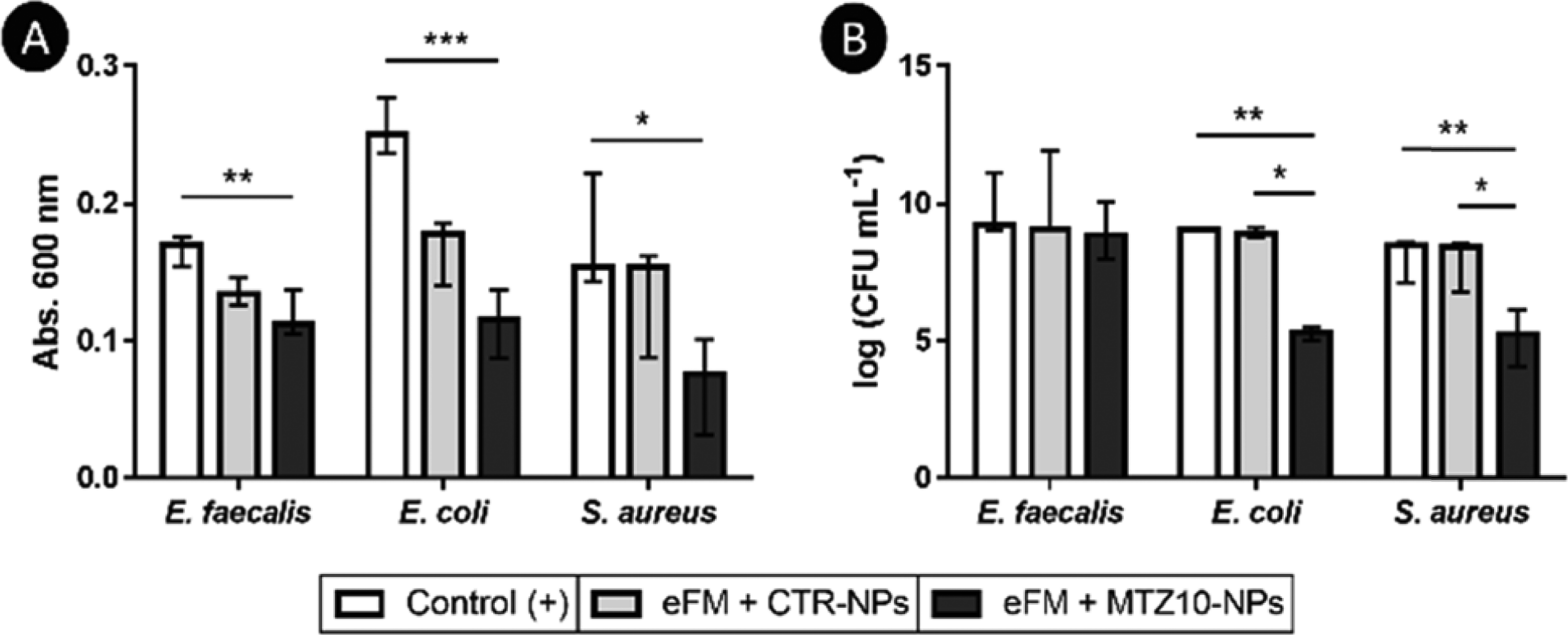
Antibacterial activity of eFMs immobilizing CTR-NPs or MTZ10-NPs for *E. faecalis*, *E. coli*, and *S. aureus*, assessed by (A) absorbance reading at 600 nm (B) or bacterial counting log (CFUs mL^–1^). Data are presented as median ± i. q. r. Results were considered statistically significant at *p* < 0.01 (*); ** *p* < 0.001; and *** *p* < 0.0001.

**Figure 9:**
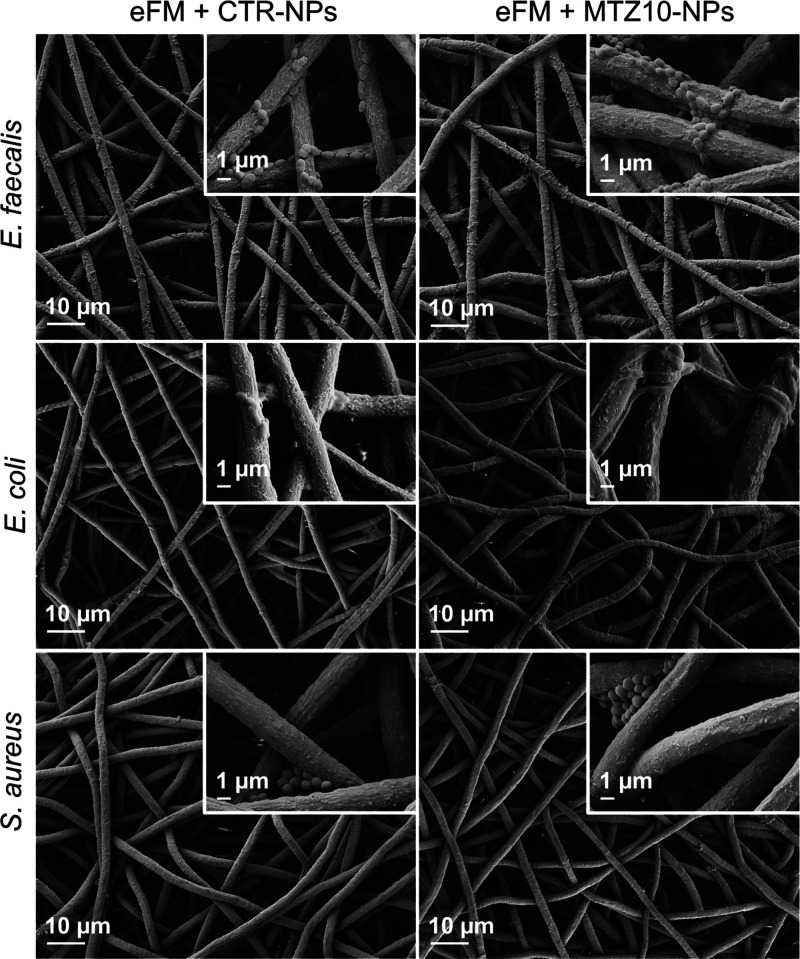
SEM observation of *E. faecalis*, *E. coli*, and *S. aureus* after 24 h of incubation with the eFM immobilizing CTR-NPs or MTZ10-NPs.

Antimicrobial tests under anaerobic conditions were carried out with eFMs immobilizing MTZ10-NPs for *E. faecalis*, *E. coli*, and *S. aureus*. Bacterial viability was quantified by measuring the absorbance at 600 nm after 24 h of incubation (Figure 8A). Significant differences were only observed between the positive control (bacteria growing in TSB) and the eFM immobilizing MTZ10-NPs (*p* < 0.001 for *E. faecalis*, *p* < 0.0001 for *E. coli*, and *p* < 0.01 for *S. aureus*). Complementarily, the results of bacterial counting (Figure 8B) demonstrated an antibacterial effect of the eFM immobilizing MTZ10-NPs against *E. coli* and *S. aureus* through a significant reduction of log_10_ CFUs mL^–1^ when compared with the positive control (*p* < 0.001) or the eFM immobilizing CTR-NPs (*p* < 0.01). No antibacterial effect was found against *E. faecalis*. The morphology of bacteria was also assessed by SEM (Figure 9). A complete disruption of the *E. coli* morphology was observed after incubation with the eFM immobilizing MTZ10-NPs in comparison with the eFM immobilizing CTR-NPs. No differences in *E. faecalis* and *S. aureus* morphologies were detected between conditions. Low retention of *E. coli* and *S. aureus* was observed in both conditions of eFMs, in contrast to *E. faecalis* that showed high retention levels.

## 4 Discussion

NPs production by the ionic gelation between the CH and TPP is an attractive technique due to its simplicity and mild process conditions. When CH comes into contact with the TPP, the polyanions can gel quickly and mediate inter- and intramolecular bonds. Briefly, electrostatic interaction occurs between the positively charged CH amine groups and the negatively charged TPP phosphate groups.^30^ Optimizing the NPs production is very important since the size distribution of NPs influences their biological performance.^19^ The polymers concentration, the proportions of CH and TPP, and pHs are among the main factors that influence the size, PDI, and zeta potential of the NPs.^19,30^ In this study, we verified that the optimum NPs size distribution was obtained at a 1:1 volume ratio of 0.3% CH:0.1% TPP solutions. At a 1:2 volume ratio, very unstable NPs were obtained due to the excess of anionic charges that promotes the precipitation or an unstable NPs formation, as reported by others.^31^ By its side, an increase of pH from 4.5 to 6 induces an increase in the size and PDI of the NPs, due to the aggregation of the NPs, as reported by others.^32^ When the pH of the solution is increased to 6, there is a greater amount of hydroxyl ions in solution, promoting bond formation with CH by deprotonation of NH_3_^+^ ions. Consequently, the CH chains bend and fewer intermolecular bonds between the CH and TPP are formed due to fewer NH_3_^+^ ions in solution.^30,33^ Mattu *et al.*(30) showed that the deprotonation of the particle surface when pH is greater than 6 promotes a lower electrostatic repulsion increasing the particle aggregation. The zeta potential has also decreased dramatically at pH 6, as expected. According to Fan *et al.*,^34^ monodisperse CH NPs are obtained at a pH ranging between 4.5 and 5.2, which was also observed in the present work. Contrary to what was reported,^30^ the pH of the TPP solution did not influence the size and PDI of the produced NPs. After optimizing all these parameters, changing the CH concentration from 0.3 to 0.2 or 0.4% and TPP concentration from 0.1 to 0.05 or 0.2% may have promoted an imbalance in the cationic charges that bond with the anionic charges, and consequently, polydisperse NPs were obtained due to the precipitation or an unstable NPs formation. This finding was more pronounced when the CH concentration was decreased or when the TPP concentration was increased. It is accompanied by a dramatical reduction in the zeta potential to slightly negative values probably as a result of insufficient cationic charges of the CH to establish the bond with the phosphate groups of the TPP. These results corroborate the findings of Sreekumar *et al.*,^31^ which show that the hydrodynamic diameter of the particles was strongly dependent on the initial CH concentration.

After optimizing the production parameters (1:1 volume ratio of a 0.3% CH concentration at pH 4.5 and 0.1% TPP – F4), the NPs were morphologically characterized, showing a solid round shape without aggregation.^19,35,36^ Other studies have shown CH NPs of a smaller size but with a higher tendency for aggregation^35^ or even larger sizes.^19,20,37^ When increasing concentrations of MTZ were used in the preparation of the NPs, there were no significant changes in size and PDI, even after long periods of storage. Likewise, there were no changes in the surface charge of the NPs. This indicates that the drug did not interfere with the intermolecular bonds between CH and TPP, maintaining the stability of NPs. Oppositely, Ruchika and Himanshi^19^ demonstrated an increase in the CH NPs size when the concentration of MTZ was increased. Sreeharsha et al.,^21^ in their stability study, reported a slight increase in particle size that may have occurred due to the attraction and agglomeration of small NPs, but the stability was maintained. Some authors have reported that an increase in the concentration of MTZ causes a decrease in the EE.^19^ In our study, neither an increase in the EE was observed with increasing concentration nor a saturation point was reached.

Previous work has developed a colon-specific delivery system based on eFMs along with CH NPs but through coaxial electrospinning, and envisioning a therapy for colon cancer.^38^ Specifically, quercetin-loaded CH NPs suspension and polyvinyl alcohol were used as the core solution, and sodium alginate and polyoxyethylene were used as the shell solution. In our work, we have intended to immobilized CH NPs at the surface of eFMs, aiming to spatially control the release of MTZ after a colorectal anastomosis. Therefore, it was required to insert oxygen-containing groups^26,29^ to enable the immobilization of NPs at eFM surfaces. The activation of the eFM by oxygen plasma allowed the binding of CH NPs to its surface. The oxygen-containing groups at the surface are, according to previous studies, able to improve surface hydrophilicity and the surface ability to establish chemical bonds.^26,39^ These results were confirmed by XPS analysis through the appearance of an additional band related to the amide bond formation and an increase on the intensity of the C–O group band in the carbon environment, as well as the detection of the N 1s curve, all characteristics of the presence of CH NPs on the eFM surface.^29,40,41^ The NPs showed a random distribution at the fibers’ surface, and their size was small enough to penetrate the pores between the fibers as reported,^35^ reaching fibers in the innermost planes of the eFMs. According to these data, the proposed strategy allowed the immobilization of NPs at the surface of the activated eFM by oxygen plasma treatment followed by the reaction with the positively charged amine/acetamide functional groups of CH, probably through a covalent bond. The contact angle measurements are in accordance with the ones previously described in the literature, where the plasma treatment significantly enhances the hydrophilicity of eFMs.^39^ Furthermore, the immobilization of NPs promoted a slight increase of the contact angle values, however without compromising the high hydrophilicity of the nanosystem.

To assess the cytocompatibility of the developed DDS, NPs loaded with different concentrations of MTZ and CTR-NPs immobilized at the surface of eFMs were seeded with human colon epithelial and fibroblastic cells. Experimental data demonstrated that an increase in MTZ concentration did not affect the cell metabolic activity, and this cytocompatibility was even increased for longer time points (7 days). Jung *et al.*(35) also proved the biocompatibility of CH NPs absorbed in eFMs previously treated with oxygen plasma. Furthermore, no changes were found in the morphology and adherence of cells to eFMs immobilizing NPs, being the cells adhered even to inner plane fibers of the eFMs. These observations are of particular importance, since they allow us to overcome the limitations related to the use of MTZ by traditional routes of administration, avoiding the unwanted and nonspecific side effects and the degradation of MTZ before reaching the colorectal environment.^13,16^ Moreover, the developed DDS also allows the use of a specific and localized concentration of MTZ, something not achieved by other routes of administration.

Since increasing the concentration of MTZ did not affect the properties of NPs (i.e., size, PDI, zeta potential, and EE) and colonic cells viability, the release profile of eFMs immobilizing NPs loaded with the higher concentration of MTZ (i.e., eFM + MTZ10-NPs) was assessed using a 0.9% NaCl solution at 37 °C, to simulate the physiological conditions of the circulating plasma that has an osmolarity of 285–295 mOsm L^–1^.^42^ In addition, the pH of the solution was adjusted to 7.4 to mimic the mild alkaline conditions of the colon.^14^ MTZ was quickly released in the first 12 h of the study, followed by a gradual release. The initial burst release can be attributed to the diffusion of the most superficial MTZ crystals and due to the hydration of the CH NPs.^19,20,37^ After this initial period, it is expected that the mechanism of MTZ release will change, and its diffusion will occur through the CH matrix. Due to the electrostatic interaction between the positively charged amino group of CH and the negatively charged phosphate group of TPP and the increase in NPs density due to the swelling of CH, the permeability to MTZ decreases. Consequently, MTZ is entrapped into NPs, promoting its slow release. Therefore, the bioavailability and, consequently, the therapeutic effect of MTZ are prolonged.^19−21^ Although Jung *et al.*(35) considered that the release of molecules will occur after the NPs detachment from the fibers, which happens after 5 days, in our study it was verified that the release of MTZ occurs right after the first moments of contact with the release medium.

After assessing the release profile of the MTZ10-NPs immobilized at the surface of the eFM, this DDS was tested against different strains of relevant Gram-positive (*E. faecalis* and *S. aureus*) and Gram-negative (*E. coli*) bacteria of the colon environment. Indeed, MTZ can act against bacteria that share an anaerobic niche in the colonic lumen or tissue abscesses.^43^ The results obtained showed that the eFM immobilizing MTZ10-NPs provided not only a reduction in *E. coli* viability through absorbance measurements but also a 4 log_10_ CFUs mL^–1^ by the viable counting method, which corresponds to a 99.99% of bacterial reduction. The morphology of *E. coli* after incubation with the eFM immobilizing MTZ10-NPs was completely disrupted when compared with the control condition (eFM + CTR-NPs), confirming the devastating effect of released MTZ. This result is especially promising, since *E. coli* is the main enteric Gram-negative rod in the colon and the most abundant bacteria after the completion of an anastomosis.^8,9^ Although some studies consider that MTZ is not activated by facultative anaerobic bacteria such as the case of *E. coli*,^13,44,45^ others had reported MTZ-mediated death.^46−50^

Usually, the colon lumen is the biggest source of contamination. However, the colorectal surgery is also affected by bacteria found on the skin and in the environment, as is the case of certain Gram-positive organisms such as *S. aureus*, also commonly associated with infections of the surgical site.^8^ Similar to *E. coli*, eFM + MTZ10-NPs also promoted a reduction in *S. aureus* viability through absorbance measurements and a viable counting method (log_10_ CFUs mL^–1^) compared to the controls. However, no differences were found in the morphology of *S. aureus* incubated with the eFM immobilizing MTZ10-NPs when compared to the eFM immobilizing CTR-NPs. Brook^51^ reported the resistance of *S. aureus* to MTZ in *in vitro* assays, but Victorelli *et al.*(52) proved a lower minimum inhibitory concentration of MTZ-loaded polyethyleneimine and CH-based liquid crystalline systems than the MTZ solution for *S. aureus* strains. Also, Bai *et al.*(49) demonstrated the antibacterial activity of a quaternary ammonium silane-grafted hollow mesoporous silica loading MTZ against *S. aureus*. For both *E. coli* and *S. aureus* suspensions, our findings confirmed that an incubation time of 24 h was sufficient to release MTZ from NPs immobilized to eFMs and promote bacterial reduction.

Regarding *E. faecalis*, the eFM immobilizing MTZ10-NPs provided a significant reduction in the absorbance measurements of bacterial viability compared to the positive control, but no significant reduction was observed in the viable counting method (log_10_ CFUs mL^–1^). Therefore, we hypothesize that the concentration of MTZ released from NPs may have promoted a temporary growth reduction or inactivation (bacteriostatic effect) in the activity of *E. faecalis* but was not enough to kill bacteria. This may occur because MTZ is released from NPs and absorbed by bacteria but fails to become active.^13,44^ Indeed, other studies have also identified an *in vitro* inactivation of MTZ by different clinical isolates.^53^ Kaushik *et al.*(54) also show that MTZ encapsulated or not within an injectable self-assembled biomimetic nanomatrix gel demonstrated no antibacterial activity against *E. faecalis*. Despite the minor reduction observed in *E. faecalis*, a very common bacterium in the colon, it is not a frequent cause of infections under normal conditions.^8^ Therefore, the developed DDS will not disrupt the normal colonic flora associated with *E. faecalis*, and a temporary inactivation of 24 h may be enough for a successful anastomosis.

Overall, the developed DDS constituted of the eFM immobilizing MTZ10-NPs showed activity against both Gram-positive and Gram-negative bacteria, but the sensitivity against *E. coli* was more pronounced. These findings were also shared by Mansour *et al.*,^55^ comparing thieno[2,3-*b*]pyridine–CH nanocomposites with thieno[2,3-*b*]pyridine derivatives. Like the NPs in the present DDS, these nanocomposites also have a positive charge that can bind to the surface of negatively charged bacteria, compromising the normal functions of their membrane.^55,56^ Another hypothesis suggested was the adsorption of NPs to the cell membrane through electrostatic interactions, which can cause precipitation and coagulation of cytoplasmic proteins followed by bacterial death.^55,57^ However, since Gram-negative bacteria present a higher negative charge on cell surfaces than Gram-positive bacteria, a stronger interaction with the NPs may occur, increasing their sensitivity comparing to Gram-positive bacteria.^55,58,59^ This situation can promote a more evident change in the bacterial morphology of *E. coli* compared to Gram-positive bacteria in which only DNA strand changes may happen due to the MTZ. Therefore, MTZ-loaded NPs immobilized at the eFM surface may act as an efficient DDS with the ability to reduce colorectal infection.

## 5 Conclusions

NPs were successfully produced by the ionic gelation technique between CH and TPP. The stability of NPs was maintained over time at a range of MTZ concentrations loaded in NPs. The different concentrations of MTZ loaded in NPs immobilized at the surface of plasma-treated eFMs did not affect the viability of colon epithelial and fibroblastic cells. This finding is of particular importance given the proposed local administration of the nanosystem, directly at the anastomotic site, reducing side effects. The MTZ10-NPs immobilized in activated eFMs showed that MTZ is released quickly within the first 12 h, being continuously and gradually released along time. This DDS also promotes a significant reduction of important bacteria involved in colorectal infection such as *E. coli* and *S. aureus*, without significantly affecting other bacteria present in the intestinal flora such as *E. faecalis*. In conclusion, the developed MTZ delivery system has high potential for preventing infections after colorectal anastomosis surgeries, without affecting the colon environment.

## Supporting Information Available

SEM images of colon epithelial and fibroblastic cells adhered to the eFM immobilizing CTR-NPs or MTZ-NPs (PDF)

## Abbreviations

CAE: constant analyzer energy
CFUs: colony-forming units
CH: chitosan
CTR-NPs: unloaded nanoparticles
DDS: drug delivery system
DMEM: Dulbecco’s Modified Eagle Medium
DMF: *N,N*-dimethylformamide
EE: entrapment efficiency
EMEM: Eagle’s Minimum Essential Medium
eFM–: electrospun fibrous meshes activated with oxygen plasma treatment
eFMs: electrospun fibrous meshes
FBS: fetal bovine serum
MTZ: metronidazole
MTZ-NPs: metronidazole-loaded nanoparticles
MTZ1-NPs: nanoparticles with 1 mg mL^–1^ metronidazole
MTZ2-NPs: nanoparticles with 2 mg mL^–1^ metronidazole
MTZ5-NPs: nanoparticles with 5 mg mL^–1^ metronidazole
MTZ10-NPs: nanoparticles with 10 mg mL^–1^ metronidazole
NPs: nanoparticles
PBS: phosphate-buffered saline
PCL: polycaprolactone
PDI: polydispersity index
SEM: scanning electron microscopy
TPP: sodium tripolyphosphate
TSA: tryptic soy agar
TSB: tryptic soy broth
XPS: X-ray photoelectron microscopy
