# Supplementary material for: Metronidazole Delivery Nanosystem Able To Reduce the Pathogenicity of Bacteria in Colorectal Infection
Source: Biomacromolecules. 2022 May 27;23(6):2415–27. doi: 10.1021/acs.biomac.2c00186 (PMC9774670; doi:10.1021/acs.biomac.2c00186)
Supplement: Supplementary file 1 — bm2c00186_si_001.pdf [file bm2c00186_si_001.pdf]

Metronidazole delivery nanosystem able to reduce the pathogenicity of bacteria in colorectal infection

Ana Oliveira[1,2,3], Ana Araújo[1,3], Luísa C. Rodrigues[1,3], Catarina S. Silva[1,3], Rui L. Reis[1,3], Nuno M. Neves[1,3], Pedro Leão[2,3], Albino Martins[1,3]*

[1]3B's Research Group, I3Bs – Research Institute on Biomaterials, Biodegradables & Biomimetics of University of Minho; Headquarters of the European Institute of Excellence on Tissue Engineering & Regenerative Medicine; AvePark - Parque de Ciência e Tecnologia, Zona Industrial da Gandra, 4805-017 Barco, Guimarães, Portugal

[2]Life and Health Sciences Research Institute (ICVS), School of Medicine, University of Minho, Campus of Gualtar, 4710-057 Braga, Portugal

[3]ICVS/3B's - PT Government Associate Laboratory, Braga/Guimarães, Portugal

*Author for correspondence: amartins@i3bs.uminho.pt

Supporting Information

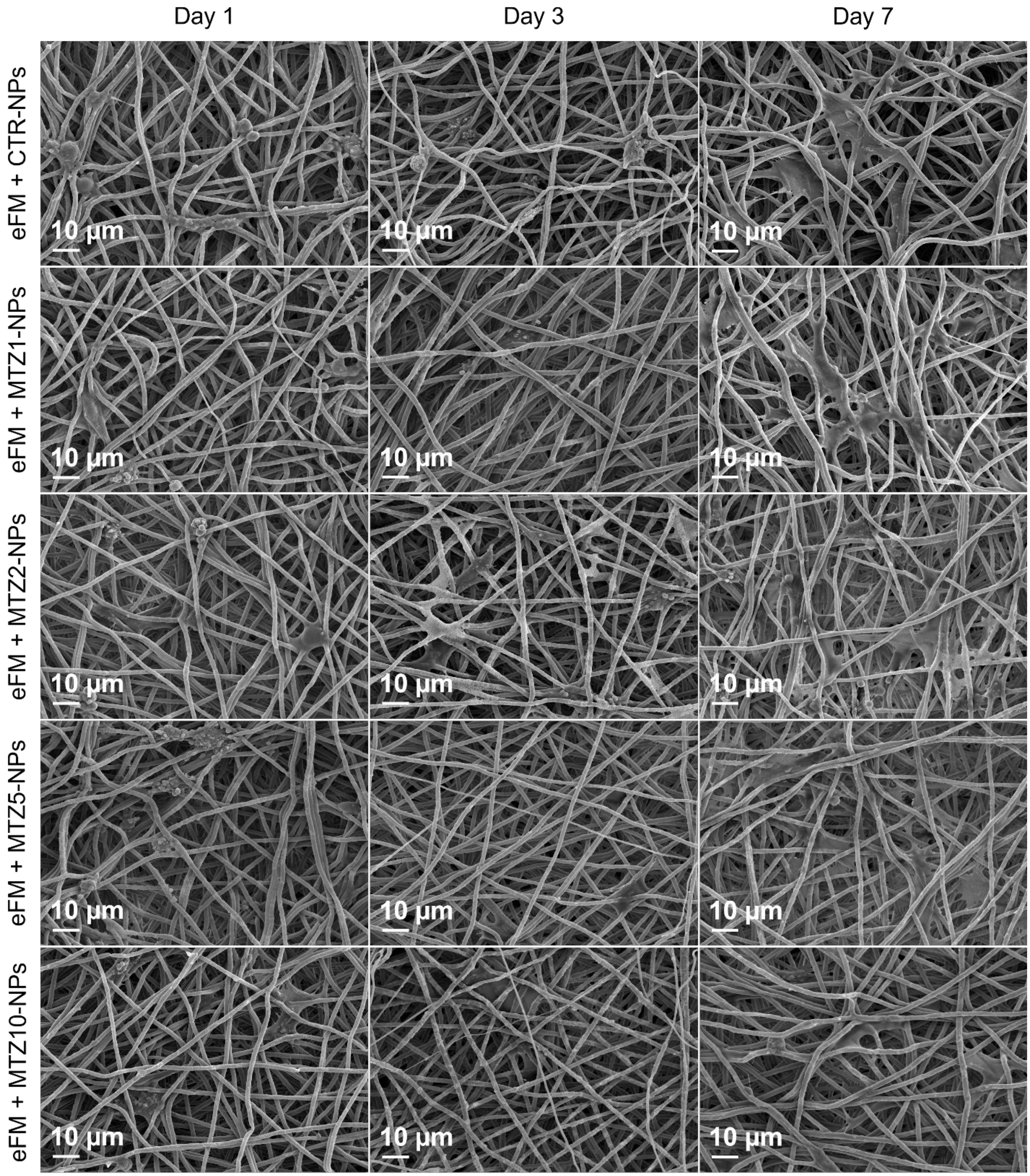

Figure S1. SEM image of colon epithelial cells adhered to the eFM immobilizing CTR-NPs or NPs loaded with different concentrations of MTZ after 1, 3, and 7 days of culture.

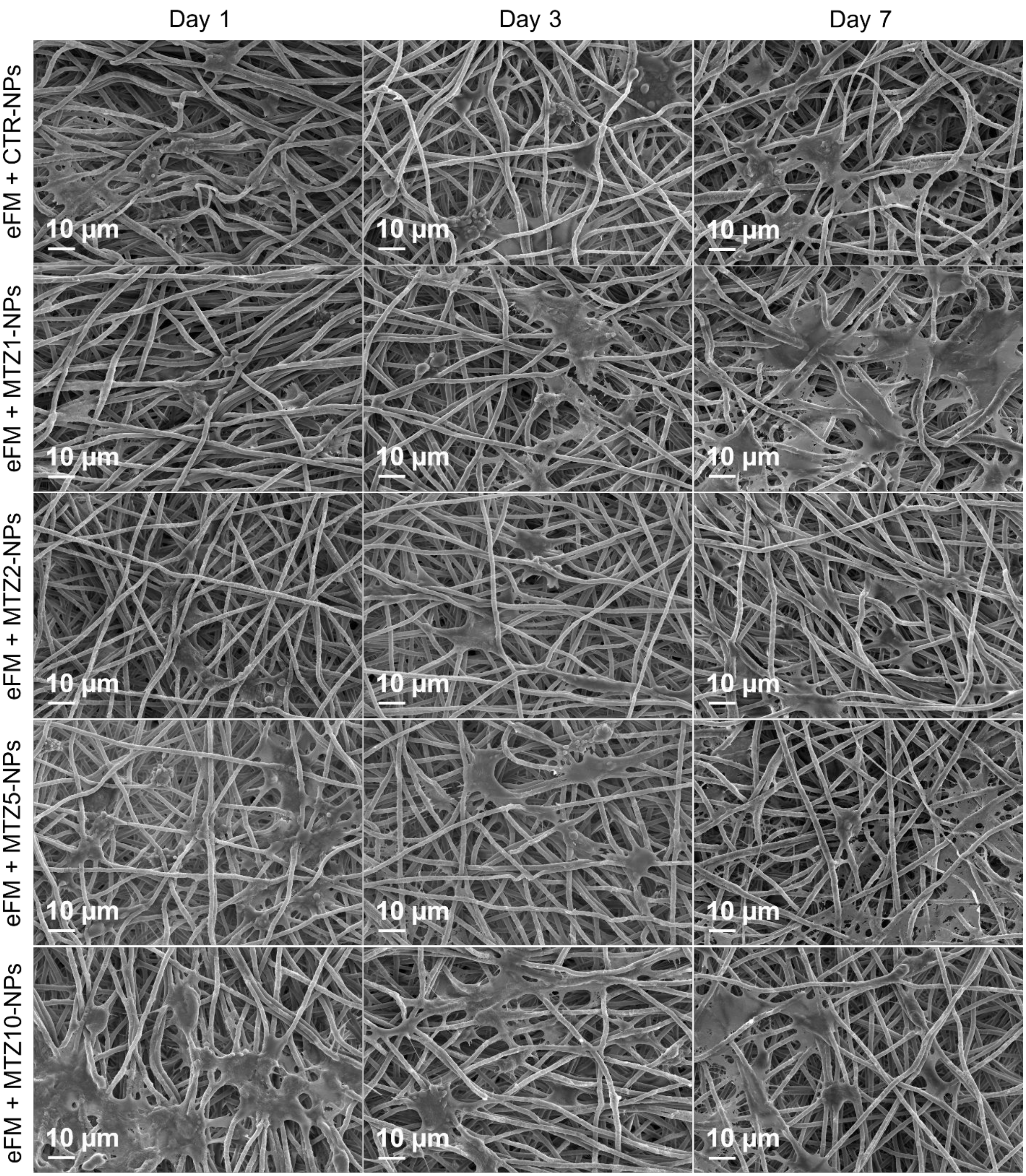

Figure S2. SEM image of colon fibroblastic cells adhered to the eFM immobilizing CTR-NPs or NPs loaded with different concentrations of MTZ after 1, 3, and 7 days of culture.
